# O06 Juvenile SLE with encephalopathy at onset

**DOI:** 10.1093/rap/rkab067.005

**Published:** 2021-10-19

**Authors:** Kishore Warrier, Vishal Paisal, Samundeeswari Deepak

**Affiliations:** Nottingham University Hospitals, Nottingham, United Kingdom

## Abstract

**Case report - Introduction:**

A 9.5-year-old girl presented with high-grade fever and rash affecting face, upper arms, chest and back with no other obvious focus. Investigations revealed pancytopaenia, raised inflammatory markers and negative cultures. CT scan of chest and abdomen revealed extensive lymphadenopathy. A diagnosis of juvenile systemic lupus erythematosus (JSLE) was made from the clinical features, including the classical rash and the laboratory profile, after ruling out oncological problems. The diagnosis was complicated by encephalopathy and macrophage activation syndrome, both of which posed therapeutic challenges, but eventually responded to the initial treatment of lupus.

**Case report - Case description:**

A previously fit and well 9.5-year-old girl of Asian origin, born of non-consanguineous relationship, presented to local hospital with history of high-grade fever (over 39 degree Celsius) and rashes for three weeks. The rash consisted of raised erythematous papules and patches affecting her cheeks, nose, upper arms, chest and back. Her initial blood tests showed anaemia, lymphopaenia and raised inflammatory markers (CRP 47mg/L). She was commenced on broad spectrum antibiotics, which were changed following growth on urine culture. She developed oral ulceration and continued to be lethargic with poor oral intake. High-grade fever persisted with dropping blood counts and inflammatory markers remaining elevated. Further microbiological and immunological (including autoantibody screen) investigations were negative. A CT scan done at this stage showed extensive axillary, mediastinal and abdominal lymphadenopathy. She was transferred to our centre for bone marrow and lymph node biopsies. Her examination revealed cervical and axillary lymphadenopathy, hepatosplenomegaly and ascites, in addition to rashes and oral ulcers. While awaiting the biopsies, she developed multiple episodes of generalised tonic clonic seizures, requiring intubation and ventilation. MRI and MRA of head did not identify an intracranial cause for the encephalopathy. Her rashes were felt to be consistent with lupus and the autoantibody profile showed strongly positive ANA (with coarse speckling pattern) and double-stranded DNA, in addition to profound hypocomplementeamia. She was also positive for Anti SM, RNP and anti-Ro52 antibodies. Her chest radiograph showed pleural effusion while echocardiogram showed global pericardial effusion. She had no proteinuria. The bone marrow and lymph node biopsies ruled out lymphoproliferative pathology. She also satisfied the EULAR (2016) criteria for macrophage activation syndrome (fever, pancytopaenia, ferritin over 32000 microg/L, elevated liver enzymes, hypertriglyceridaemia and hypofibrinogenaemia). The encephalopathy was thought to be due to macrophage activation syndrome (MAS).

**Case report - Discussion:**

Although her presentation with fever and pancytopaenia with subsequent discovery of extensive lymphadenopathy raised the possibility of lymphoproliferative pathology, the dermatology opinion was clearly favouring JSLE in view of the classical rash. The mouth ulcers, serositis, haematological and immunological profile all supported the diagnosis. She satisfied the clinical and laboratory criteria for MAS, which was thought to be the cause of her encephalopathy too, as there were no changes to suggest lupus or vasculitis, or any other pathology like vascular events or posterior reversible encephalopathy syndrome (PRES), on neuroimaging. She was started on pulse of intravenous methyl prednisolone, which was continued for a further two days due to MAS, followed by cyclophosphamide at 500mg/m2. The plan is to continue cyclophosphamide to induce remission, in addition to slow weaning course of oral steroids and hydroxychloroquine. She had an excellent clinical and laboratory response at the first review since discharge.

**Case report - Key learning points:**

1) The initial diagnosis with fever and extensive lymphadenopathy was challenging, although it has been reported in the literature as more common in childhood-onset lupus, when compared to lupus presenting in adulthood. The multi-disciplinary team working involving the rheumatology, dermatology, neurology and oncology teams made the diagnostic process more efficient and timely.

2) The diagnostic dilemma over the cause of encephalopathy with neuroimaging suggestive of no changes suggestive of lupus, vasculitis, vascular events or other pathology like PRES (although the seizures occurred prior to use of steroids and she had no evidence of hypertension or renal involvement). Although difficult to prove, we put this down to MAS and she had a significant clinical improvement, in line with improvement of laboratory parameters of MAS.

3) Her MAS settled down with steroids, without having to resort to alternate agents like cyclosporine or anti-interleukin1 agents. The choice of agent to treat MAS, had it not settled down with the initial pulse of steroids, was debated – we would have had to choose an agent that may not be as effective in the management of lupus. We were not comfortable with choosing two different agents to treat the condition and a complication of the condition; but the extended course of steroids worked well for both.

